# Antibiotic Abuse in Ornamental Fish: An Overlooked Reservoir for Antibiotic Resistance

**DOI:** 10.3390/microorganisms13040937

**Published:** 2025-04-18

**Authors:** Chun Au-Yeung, Yat-Lai Tsui, Man-Hay Choi, Ka-Wai Chan, Sze-Nga Wong, Yuk-Ki Ling, Cheuk-Ming Lam, Kit-Ling Lam, Wing-Yin Mo

**Affiliations:** 1 Department of Applied Science, School of Science and Technology, Hong Kong Metropolitan University, Ho Man Tin, Kowloon, Hong Kong; auyeungc@hkmu.edu.hk (C.A.-Y.); s1289007@live.hkmu.edu.hk (Y.-L.T.); mhchoi@hkmu.edu.hk (M.-H.C.); chankw@hkmu.edu.hk (K.-W.C.); sznwong@hkmu.edu.hk (S.-N.W.); ykling@hkmu.edu.hk (Y.-K.L.); chmilam@hkmu.edu.hk (C.-M.L.); kllam@hkmu.edu.hk (K.-L.L.); 2 Department of Food Science and Nutrition, Faculty of Science, The Hong Kong Polytechnic University, Hung Hom, Kowloon, Hong Kong

**Keywords:** antibiotics, antibiotic resistance, zoonotic pathogens, One Health, good aquaculture practices

## Abstract

Ornamental fish represent a significant aquaculture sector with notable economic value, yet their contribution to antibiotic residues and resistance remains underrecognized. This review synthesizes evidence on widespread and often unregulated antibiotic use—including tetracyclines and fluoroquinolones—in ornamental fish production, transportation, and retail, primarily targeting bacterial diseases such as aeromonosis and vibriosis. Pathogenic microorganisms including *Edwardsiella*, *Flavobacterium*, and *Shewanella* spp. cause diseases like hemorrhagic septicemia, fin rot, skin ulcers, and exophthalmia, impairing fish health and marketability. Prophylactic and therapeutic antibiotic applications elevate antibiotic residues in fish tissues and carriage water, thereby selecting for antibiotic-resistant bacteria (ARB) and antibiotic resistance genes (ARGs). These resistant elements pose significant risks to fish health, human exposure via direct contact and bioaerosols, and environmental health through contamination pathways. We emphasize the urgent need for a holistic One Health approach, involving enhanced surveillance, stringent regulatory oversight, and adoption of alternative antimicrobial strategies, such as probiotics and advanced water treatments. Coordinated global actions are crucial to effectively mitigate antibiotic resistance within the ornamental fish industry, ensuring sustainable production, safeguarding public health, and protecting environmental integrity.

## 1. The Ornamental Fish Industry: An Overview

The ornamental fish trade is a thriving global industry, with over 4500 freshwater species and 1450 marine species traded worldwide in 2010 [1]. In 2009, estimates of the total value of the industry were at USD 15–30 billion and involved more than 1.5 billion live ornamental fish traded per year [2,3]. Ninety nine percent have been for hobbyist aquaria, with the remaining 1% destined for public aquaria or research laboratories since 2004 [4]. Due to the increasing popularity of keeping home aquaria over recent decades, a substantial increase in trade and demand for ornamental fish has occurred, with the trade growing by 14% annually since the 1970s [5].

**Figure 1:**
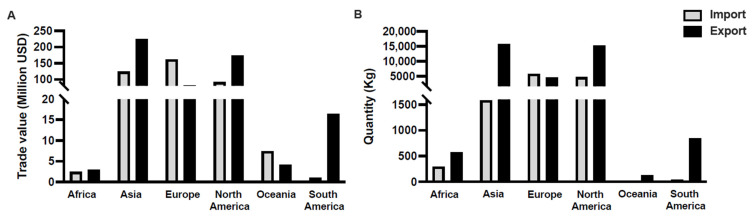
(**A**) Trade value and (**B**) quantity of live ornamental fish trade by continent in 2023.

Global exports have increased from USD 180 million in 2000 to USD 337 million in 2023 [6,7]. The trade spans more than 125 countries, highlighting its global scale [8]. As shown in Figure 1A, in 2023, Asian countries were the dominant exporters, accounting for 66.34% of the trade with exports valued at USD 224 million [7]. Among Asian exporters, Japan, Indonesia, Singapore, Sri Lanka, Thailand, Malaysia and Myanmar together contributed 40.42% of the region’s export market. China and Hong Kong ranked among the top 20 exporters, contributing approximately USD 5 million to the trade [7]. Europe followed as the second-largest exporting region, accounting for 24.29% of total exports (valued at USD 82 million), followed by South America (16.45%), North America (8.08%), Oceania (4.11%) and Africa (2.98%) [7].

In 2023, Europe emerged as the largest global trade bloc for ornamental fish, accounting for 41.63% of the market [9]. Asia followed with 31.92%, North America with 23.65%, Oceania with 1.91%, Africa with 0.63%, and South America with 0.26%. The United States remained the world’s largest single market for ornamental fish, accounting 21.19% of imports, valued at USD 82 million. Within Europe, the United Kingdom, Germany, France, The Netherlands, Spain, and Italy were the leading importers, collectively making up 16.45% of the global trade, valued at USD 70 million. In Asia, China–Hong Kong leads imports at 12.53% (valued at USD 49 million), yet this thriving trade obscures a growing concern: antibiotic abuse.

According to the Food and Agriculture Organization of the United Nation (FAO), the global ornamental fish trade utilized about 4 million tons of fish [10]. As shown in Figure 1B, in 2023, the global export volume of live ornamental fish reached around 37,000 tons, with Asia contributing the largest share (70.17%), followed by Europe (20.44%), South America (3.77%), Africa (2.56%), North America (2.46%), and Oceania (0.59%) [7]. During the same period, Europe led global imports at 46.83%, followed by North America and Asia, while Africa, South America, and Oceania collectively accounted for only 2.87% of total imports [9]. Among the most popular freshwater families in the ornamental fish industry (Table 1) are Characidae, Cichlidae, Cyprinidae, Gasteropelecidae, Loricariidae, and Poeciliidae [11]. Notably, the Poeciliidae and Characidae families accounted for over a quarter of the market share by volume [6].

## 2. Antibiotic Use and Resistance in Ornamental Fish

The ornamental fish industry is a relatively underexplored subsector of aquaculture, but it plays a significant role in the emergence and spread of antibiotic resistance. The global ornamental fish trade relies on extensive transnational supply chains, transporting fish sourced or raised in diverse exporting regions to aquarists worldwide. These supply chains are intricate, often involving multiple intermediaries, such as exporters, importers, distributors, wholesalers, and retailers, before the fish reach their final destination [12]. Ensuring the survival of ornamental fish throughout the complex supply chain poses a critical challenge, as stressors actively compromise their health and elevate mortality rates. Stressors encountered by ornamental fish during the supply chain include frequent human handling, restricted living space, high stocking density, mechanical disturbance during transport, suboptimal water quality, accumulation of harmful waste products, and reduced dissolved oxygen levels [11,12]. Prolonged exposure to stressors weakens the immune systems of fish, leading to immunosuppression and heightened susceptibility to infections, eventually increasing rates of morbidity and mortality [13,14]. Prevalent diseases in this sector often cause substantial losses in ornamental fish production, affecting husbandry and research facilities, with cumulative mortality rates reaching up to 60% in some cases [15,16,17].

Other than “dead or alive”, the appearance of ornamental fish also plays a crucial role in determining their market value. Unlike fish bred for food production or recreational purposes, ornamental fish are primarily valued for their aesthetic qualities [18]. Many common bacterial diseases significantly impair the aesthetic quality of ornamental fish. For example, infections caused by *Aeromonas* spp. can lead to severe scale loss, tail rot, and body ulcers, reducing their visual attractiveness [19]. Similarly, *Shewanella* and *Aeromonas* spp. are associated with exophthalmia (“pop-eye”) and corneal opacity, which distort eye clarity and overall appearance in various ornamental species [20]. Signs of disease or infection can significantly reduce consumer willingness to purchase fish, as aquarists are concerned not only about aesthetics but also about introducing pathogens into their existing aquarium systems. This concern creates a strong economic incentive for sellers to use antibiotics prophylactically or therapeutically to treat infections before selling fish to aquarists, contributing to the widespread use of antibiotics in the ornamental fish trade [21].

### 2.1. Antibiotic Practices in the Industry

Antibiotics are widely used in the ornamental fish industry during husbandry, transportation, and at retail shops [22]. Industry practitioners primarily use antibiotics to reduce infections that occur under stressful conditions, where transportation alone can result in mortality rates as high as 30% [8]. Antibiotics are commonly given via oral administration or bath treatments, used for both therapy and prophylaxis [22]. Practitioners incorporate antibiotics into food or deliver them directly via a rubber feeding tube through the mouth into the stomach or intestines [23]. Medicated feeds, the most common administration method, involve preparing flake-, pellet-, or gel-based diets infused with antibiotics, such as oxytetracycline at 55–83 mg/kg/day for 10 days for Gram-negative bacterial infections and erythromycin at 100 mg/kg/day for 14 days for Gram-positive infections [24,25]. Alternatively, bath treatments apply antibiotics directly to an aquarium or a separated aquarium to allow absorption through the gills and skin [23]. For example, experts recommend using oxytetracycline at 100–400 mg/L for 1 h daily over 10 days or enrofloxacin at 2.5–5 mg/L for 5 h daily over 7 days [25]. However, farmers and retailers often use these antibiotics without veterinary consultation, raising concerns about antibiotic resistance, environmental contamination, and diminished treatment efficacy.

Researchers have rarely examined antibiotic use in the ornamental fish industry in-depth. To our knowledge, most existing research focuses on indirect evidence, such as detecting ARB and antibiotic resistance genes (ARGs) in ornamental fish and their carriage water (water used for transport, storage, and maintenance), indirectly confirming antibiotic use. This water frequently contains fish waste, uneaten food, and microorganisms, making it a reservoir for ARB. Unlike the food fish aquaculture sector, which is subject to more stringent antibiotic regulations in many countries, the ornamental fish industry suffers from minimal oversight. While food fish production requires U.S. Food and Drug Administration (FDA) approval for antibiotics such as oxytetracycline, florfenicol, sulfamerazine, and sulfadimethoxine/ormetoprim, the ornamental fish industry employs a broader range of antibiotics, including fluoroquinolones, tetracyclines, and nitrofurans [12,26,27]. Studies show most globally exported ornamental fish carry antibiotic residues, contributing to bacterial resistance against drugs such as oxalinic acid, oxytetracycline, chlortetracycline, sulfamethoxazole/trimethoprim, and chloramphenicol [28,29,30,31,32]. Research has also demonstrated that antibiotic concentrations in the environments of ornamental fish markets rival those in treated effluent from sewage treatment facilities, further raising concerns about the emergence and spreading of antibiotic resistance in the industry [32,33].

As summarized in Table 2, studies have reported significant levels of antibiotics in the carriage water of ornamental fish. For example, our previous studies measured oxytetracycline and tetracycline concentrations up to 2.26 mg/L and 0.21 mg/L, respectively [28,29]. Compared to a study in northern China, tetracycline concentrations in carriage water were 820 times higher than in wastewater from a local ornamental fish market, indicating substantial tetracycline abuse [32]. Frequently detected antibiotics in the ornamental fish industry include sulfamethoxazole, chlortetracycline, doxycycline, ciprofloxacin, enrofloxacin, clarithromycin, and roxithromycin [32,34]. Residual levels of quinolones, chloramphenicol, and nitrofurans have also been measured, reaching up to 6.3 mg/L, 13 mg/L, 0.04 mg/L, and 0.039 mg/L, respectively [30]. The half-lives of antibiotics in freshwater environments vary, with some persisting for extended periods. For example, the half-life of oxytetracycline in freshwater is about 58 h, while that of ciprofloxacin ranges from 212 to 378 h [35,36]. As a result, certain antibiotics can persist in aquatic environments throughout the supply chain.

The persistence of antibiotics hinders efforts to monitor and manage residues over time, leaving notable gaps in the understanding of their effects on fish health and aquarium ecosystems. These residues allow ARB to outcompete susceptible strains by enhancing their ability to acquire nutrients and occupy available niches, which leads to shifts in bacterial community composition [37,38]. While high antibiotic concentrations often inhibit susceptible strains, continuous introduction of sub-inhibitory levels of antibiotics significantly increases the abundance of ARGs and integrase gene *intl*1 in microbial communities [37,39,40,41]. Haenen et al. confirmed the presence of significant antibiotic residues in the carriage water of ornamental fish from regions or countries such as Brazil, China, Hong Kong, Singapore, Sri Lanka, and Thailand, highlighting the widespread use of antibiotics throughout the supply chain [30]. Taken together, these findings emphasize a gap in the monitoring of antibiotic use and managing antibiotic residues within the ornamental fish industry to mitigate the spread of antibiotic resistance. Beyond antibiotic use patterns, bacterial infections in ornamental fish further exacerbate resistance risks.

### 2.2. Bacterial Diseases and Resistance in Ornamental Fish

Ornamental fish are susceptible to a wide range of diseases caused by opportunistic bacteria, which are often considered part of their normal flora. These bacteria play a critical role in disease outbreaks, particularly when environmental conditions deteriorate, or fish experience stress. Notably, even in asymptomatic fish, opportunistic bacterial isolates often display multidrug resistance, challenging antibiotic resistance management [42].

Bacterial infections can result in severe morbidity and mortality, ultimately impacting both fish health and industry productivity. Common bacterial diseases affecting ornamental fish include aeromoniasis, flavobacteriosis, mycobacteriosis, nocardiosis, pasteurellosis, streptococcosis, and vibriosis [35]. As summarized in Table 2, Gram-negative bacteria are natural inhabitants of the aquatic environments of ornamental fish and are the primary causative agents of infections in the industry, while Gram-positive bacterial infections are relatively less common [43,44].

**Figure 2:**
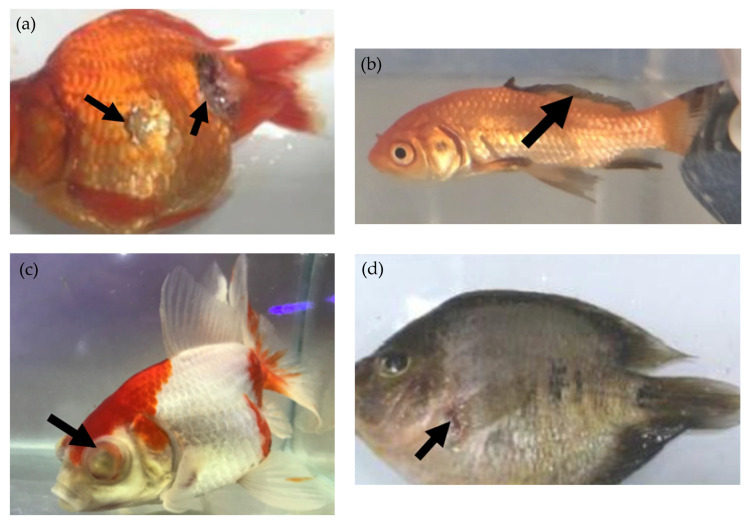
Photographs of ornamental fish exhibiting bacterial diseases: (**a**) skin ulceration; (**b**) fin rotting; (**c**) exophthalmia; (**d**) hemorrhage [20,65]. Arrows indicate the affected areas.

Gram-negative fish pathogens, such as *Aeromonas*, *Vibrio*, and *Edwardsiella* spp., are particularly damaging in ornamental fish breeding farms [15,17,45]. For example, aeromoniasis, caused by *Aeromonas* spp. (including *A. caviae*, *A. hydrophila*, *A. sorbria*, and *A. veronii*), primarily affects freshwater species such as goldfish (*Carassius auratus*), guppies (*Poecilia reticulata*) and koi carp (*Cyprinus carprio*), often resulting in hemorrhagic septicemia and ulcerative skin lesions [46]. In vivo studies have demonstrated that *A. veronii* can cause 100% mortality in goldfish and koi carp within one week [15,46,47]. Flavobacteriosis, which is caused by *Flavobacterium columnare*, leads to gill necrosis, fin erosion, and rapid mortality in various ornamental fish species, including goldfish, koi carp, molly (*Poecilia latipinna*), and platies (*Xiphophorus maculatus*) [48,49,50]. Vibriosis, induced by *Vibrio* species, causes skin ulceration, hemorrhages, and damage to internal organs in goldfish, molly, and koi carp [51,52]. Mycobacteriosis and Nocardiosis, caused by atypical nontuberculous *Mycobacterium* (NTM) and *Nocardia* species, respectively, are chronic granulomatous infections commonly observed in ornamental fish [53,54]. NTM species, including *Mycobacterium marinum*, *M. foruitum*, and *M. chelonae*, are known to infect ornamental fish such as goldfish, guppies, and zebrafish (*Denio rerio*), causing chronic wasting and organ damage [44,55,56,57]. Streptococcosis, caused by *Streptococcus* spp., infects ram cichlids (*Mikrogeophagus ramirezi*), bala sharks (*Balantiocheilos melanopterus*) and rainbow sharks (*Epalzeorhynchos frenatum*), and manifests as neurological disorders, hemorrhages, and eye lesions, especially in high-density aquaculture settings [58,59]. Figure 2 shows photos of ornamental fish exhibiting common bacterial diseases.

In addition, research has reported that *Aeromonas* spp. exhibits high resistance rates to antibiotics such as penicillin, quinolones, and tetracyclines [42,46]. Similarly, infections caused by *Vibrio* spp., including *V. cholera*, *V. mimicus*, and *V. vulnificus*, may result in severe mortality. Infections caused by *V. cholera* showed 100% mortality in koi carp within one week [15]. Details of ARB exhibiting resistance to antibiotics have been described by Ruppé et al., which are either through the expression of enzymes that inactivate those antibiotics, or through non-enzymatic mechanisms [60]. Antibiotic resistance in *Vibrio* spp. is also concerning, with studies reporting a multiple antimicrobial resistance (MAR) index of 0.33 for *V. vulnificus*, indicating high resistance levels in strains isolated from the carriage water of ornamental fish [51].

*Edwardsiella* spp., particularly *E. tarda*, infect diverse fish species across wide regions and pose a notable zoonotic risk [61]. *E. tarda* is naturally resistant to several antibiotics commonly used in the ornamental fish industry, such as macrolides and benzylpenicillin [62]. Isolates from a goldfish farm with a 60% cumulative mortality rate have exhibited resistance to multiple antibiotic classes, including β-lactams, cephalosporins, aminoglycosides, chloramphenicol, tetracyclines, macrolides, and sulfonamides [17]. Infections caused by *Citrobacter freundii* are also severe, with the bacterium showing resistance to penicillin, cephalosporin, tetracycline, sulfonamide, and aminoglycoside antibiotics in various ornamental fish [20,63].

While Gram-negative bacteria dominate as pathogens in diseased ornamental fish, Gram-positive bacteria, such as *Streptococcus* spp., also contribute to infections. Although less common, *Streptococcus* spp. infections can cause significant morbidity, particularly in stressed fish or those exposed to poor water quality that weakens their immune systems [58]. Under such conditions, these typically commensal or environmental bacteria can become opportunistic pathogens. This situation highlights the importance of maintaining optimal aquaculture practices, including rigorous water quality management and stress control, to prevent outbreaks of both Gram-negative and Gram-positive bacterial infections.

Moreover, resistance in NTM species is also concerning; more than 15% of isolates from ornamental fish are resistant to antibiotics such as clarithromycin, sulfamethoxazole, ciprofloxacin, and doxycycline [64]. Particularly troubling is that the minimal inhibitory concentration (MIC) of ciprofloxacin for these isolates exceeds levels typically achievable in vivo, which suggests that drug resistance to human-use antibiotics in the ornamental fish industry poses a significant public health risk [64]. Thus, the high prevalence of fish diseases and bacterial infections, coupled with the emergence of multidrug-resistant pathogens, presents a significant challenge to the ornamental fish industry.

### 2.3. Antibiotic Resistance Genes

The occurrence of antibiotic resistance genes (ARGs) in the aquatic environments of ornamental fish provides further evidence of the growing challenge of antibiotic resistance in this industry. Antibiotic use in ornamental fish farming promotes ARGs in fish bacteria, which can transfer resistance to zoonotic species through horizontal gene transfer (HGT) via mobile genetic elements (MGEs) like plasmids and integrons [66]. Resistance genes are often associated with MGEs, which facilitate their transfer between different bacterial genera and species through HGT mechanisms such as conjugation [67]. Integrons and plasmids are particularly critical for HGT, as they enhance the mobility of ARGs and accelerate their dissemination across diverse microbial populations [68]. For example, tetracycline resistance genes (*tet*E and *tet*A) and quinolone-resistance resistance genes (*qnr*S) are predominantly plasmid-mediated, whereas sulfonamide-trimethoprim-resistant resistance genes (*sul*1 and *dfr*A) are integron-mediated [68]. Research conducted by Dobiasova et al. demonstrated the transfer of *qnrS2* and *aac(6′)-Ib-cr* harboring plasmids among *Aeromonas* spp. in aquatic environments, underscoring the critical role of MGEs in propagating resistance genes [69].

Such transmission pathways are particularly concerning in the ornamental fish industry, where dense microbial populations and frequent antibiotic use create hotspots for ARG exchange. Sub-inhibitory concentrations of antibiotics exacerbate this issue by activating recombinases, such as integrases and transposases, thereby enhancing the mobility of ARGs [37]. For example, subtherapeutic antibiotic treatments strongly induce the expression of genes mediating the conjugative transfer of IncU plasmids [70]. These MGEs enable ARGs to persist and disseminate in aquatic environments, even in the absence of selective pressure, compounding their long-term impact on public and environmental health [71]. Additionally, biofilms on surfaces of aquarium filters may serve as critical reservoirs for ARGs. These biofilms provide a stable niche where bacteria can exchange genetic material, thereby enhancing the persistence of ARGs under environmental stress [72,73]. Given the scarcity of data on antibiotic use in the ornamental fish industry, the detection of ARGs has become a key method for evaluating antibiotic resistance in these environments.

Table 3 summarizes the most prevalent ARGs detected across various antibiotic classes in ornamental fish and their related environments. Tetracycline resistance genes, such as *tet*A, *tet*B, *tet*C, *tet*D, and *tet*E, are among the most frequently detected, with 40% of *Aeromonas* spp. isolates from koi carp and common carp carrying these ARGs [74]. Beta-lactam resistance genes, such as *bla*_TEM_, were widely detected in ornamental fish farms, with the highest prevalence across all fish samples [75,76]. Sulfonamide resistance genes, particularly *sul*1 and *sul*2, are found in high concentrations in wastewater and carriage water samples associated with ornamental fish [32]. Plasmid-mediated quinolone resistance (PMQR) genes, including *qnr*S2, *qnr*B17, and *aac(6′)-Ib-cr*, were identified in 19% of koi carp isolates and 24% of ornamental fish isolates, with *qnr*S2 being the most prevalent [69]. Chloramphenicol resistance genes, like *flo*R, were detected in 17.2% of bacterial isolates and in 85.7% of microbial DNA samples from carriage water, indicating their widespread occurrence [77]. Compared to other antibiotic classes, macrolide resistance determinants, such as *ere*A and *mph*A, are less prevalent but still contribute to the overall diversity of ARGs in ornamental fish environments [75,76].

The presence of ARGs linking ornamental fish to clinical settings heightens public health concerns. Table 4 summarized selected antibiotic class commonly used and associated resistance genes in ornamental fish and its carriage water. Although direct evidence of such transfer is limited, research have revealed high genetic similarity in ARGs and MGEs between environmental bacteria and clinically relevant pathogens, highlighting the role of aquatic environments as reservoirs for ARGs such as the *qnr* and extended-spectrum β-lactamase (CTX-M), which can transfer to clinical pathogens [78,79]. Consequently, the potential dissemination of ARGs from ornamental fish environments to human pathogens should be considered as a critical risk. This underscores the urgent need for further investigation and monitoring to better understand and mitigate the public health impacts of antibiotic resistance on the ornamental fish industry. Future research could explore the prevalence of antibiotic-resistant zoonotic pathogens in household aquaria and assess the efficacy of interventions, such as water filtration or hygiene protocols, to mitigate human exposure risks.

**Table 3:** Common bacterial diseases in freshwater ornamental fish: causative agents, symptoms, and hosts.

Category	Bacterial Genus	Diseases	Symptoms	Host Range	References
Gram-negative bacteria	*Aeromonas* spp.	Aeromonosis	Sloughing of scales, skin, fins, abdominal dropsy, hemorrhage.	Wide range of ornamentals	[19,46,80]
	*Citrobacter* spp.	Granulomatous diseases, meningitis	Lethargy, hemorrhagic septicemia, enteritis exophthalmia, and bleeding in eyes.	Angelfish, doctor fish, stingray	[81,82,83]
	*Edwardsiella* spp.	Edwardsiella septicemia	Discolored skin patches, external and enteric septicemia, erratic swimming, exophthalmia.	Koi, goldfish, zebrafish, and catfishes.	[84,85,86]
	*Flavobacterium* spp.	Flavobacteriosis	Yellowy white filamentous lesions predominantly affecting skin, gills, and fins.	Wide range of freshwater ornamentals	[49,50,87,88,89]
	*Klebsiella* spp.		Skin lesion	*Cyprinus carpio*	[90]
	*Vibrio* spp.	Vibriosis	Ulceration and hemorrhagic septicemia.	Wide range of ornamentals	[44,51,52]
	*Pseudomonas* spp.	Pseudomoniasis	Tail and fin rot, hemorrhage, and ulceration.	Wide range of ornamentals	[91]
Gram-positive bacteria	*Streptococcus* spp.	Streptococcosis	Hemorrhages, exophthalmia, ulceration, and erratic spiral swimming	Cyprinids and cichlids, such as red-tail black sharks, rainbow sharks, oscar, and blueram	[58,59,92]
Acid-fast bacteria	*Mycobacterium* spp.	Mycobacteriosis	Ulceration, swollen abdomen, and exophthalmia	Most ornamentals	[93,94]
	*Nocardia* spp.	Nocardiosis	Presence of granulomas in skin, muscles and various internal organs.	Neon tetras, oscar, goldfish, and Chinese high-fin banded sharks	[53,54]

**Table 4:** Selected antibiotic class commonly used and associated resistance genes in ornamental fish and its carriage water.

Antibiotic Class	Related Antibiotic Resistance Genes											
Beta-lactam			*bla*_OXA1_	*bla*_DHA1_, *bla*_TEM_	*bla*_P1_ ^#^					*bla*_TEM-1_, *bla*_OXA7_		
Chloramphenicol			*flo*R	*cat*, *cm*A1, *flo*R	*cat*B3 ^#^, *cml*A1 ^#^, *cml*A2 ^#^			*cml*A, *flo*R, *cfr*		*flo*R		
Macrolide- lincosamide-streptogramine				*mph*A	*ere*A ^#^							
(Fluoro) quinolone			*qnr*S2, *qnr*B17, *aac(6′)-Ib-cr*		*qnr*VC1 ^#^		*qnr*S, *qnr*B, *aac(6′)-Ib-cr*	*qnr*B, *qnr*S, *qep*A	*qnr*A, *qnr*S	*qnr*S, *qnr*S2		*qnr*B, *qnr*S
Sulfonamide /Trimethoprim		*sul*1, *dfr*A17 ^#^	*sul*1, *sul*2	*dfr*A12, *dfr*A14, *sul*1, *sul*2	*dfr*2d ^#^, *dfr*16 ^#^, *dfr*A1 ^#^, *dfr*A5 ^#^, *dfr*A12 ^#^, *dfr*A15 ^#^, *dfr*A21 ^#^, *dfr*A22 ^#^, *dfr*A27 ^#^, *dfr*A32 ^#^, *dfr*B3 ^#^, *dhfr*XVb ^#^, *sul*1, *sul*2			*sul*1, *sul*2, *sul*3		*sul*1, *dfr*12, *dfr*13, *dfr*A1		
Tetracycline	*tet*A, *tet*D, *tet*E		*tet*A, *tet*B, *tet*D, *tet*E	*tet*A	*tet*A, *tet*B, *tet*G		*tet*A, *tet*E	*tet*A, *tet*B, *tet*M, *tet*O, *tet*W	*tet*A, *tet*C	*tet*D, *tet*E, *tet*G	*tet*A, *tet*B, *tet*M, *tet*S, *tet*W	*tet*A, *tet*B, *tet*E
Integron	*intl*1	*intl*1				*intl*1, *intl*2	*intl*1	*intl*1, *intl*2	*intl*1	*intl*1		*intl*1
References	[74]	[95]	[69]	[75]	[76]	[96]	[97]	[32]	[41]	[77]	[98]	[99]

^#^ integron gene cassettes related.

## 3. Human Health Risks from Ornamental Fish Keeping

While substantial evidence exists regarding the use of antibiotics and proliferation of antibiotic resistance in ornamental fish, the direct links between resistance in these aquatic organisms and human health impacts remain incompletely understood. On the one hand, aquarists are well aware of the potential harm posed by chemicals and drugs associated with ornamental fish. For example, experienced aquarists routinely discard the carriage water used during fish transportation—a practice rooted in the awareness that this water may carry residual chemicals, drugs, and potentially harmful bacteria. By disposing of this water immediately, they aim to reduce the risk of contaminating household aquaria with microorganisms and chemical residues. Moreover, it is widely understood that ornamental fish are not intended for human consumption. Although occasional social media posts depicting cooked ornamental fish have prompted organizations like the Hong Kong Centre for Food Safety to issue public reminders about this distinction, the consumption of ornamental fish—especially those that are also considered edible—can still pose potential health risks through exposure to prophylactic drugs and chemicals [100].

On the other hand, there remains a significant gap in our understanding of the direct health risks to household aquarists, particularly relating to the selection and spread of antibiotic resistance. Previous literature consistently indicates that antibiotic use in the ornamental fish sector could exacerbate antimicrobial resistance, with potentially severe health implications for humans [101,102]. This concern is heightened by the abuse of antibiotics in the ornamental fish trade, especially through transboundary and international movement, which may facilitate the translocation of ARB and ARGs. Despite these warnings, no specific conclusions regarding direct human health risks have been established. Bridging this knowledge gap is crucial to clarify how potential risks continue to persist within household aquaria, ultimately impacting aquarists and possibly broadening the public health challenge of antimicrobial resistance.

### 3.1. Sources of Antibiotic Residues in Aquaria

When bacteria are not exposed to antibiotics, their antibiotic resistance tends to gradually decrease, as demonstrated by Lázár et al. [103]. This occurs because maintaining ARGs places a metabolic burden on bacteria, resulting in disadvantages such as reduced growth rates and diminished transmission capabilities [104,105]. Based on this understanding, it would be reasonable to expect that ARB associated with ornamental fish would gradually lose their resistance when maintained in an antibiotic-free environment. Achieving antibiotic-free conditions in aquaria is challenging due to continuous antibiotic introduction from multiple sources. This constant presence of antibiotics in household aquarium systems makes it both difficult and costly to establish truly antibiotic-free conditions, thereby sustaining ARB populations rather than allowing for their natural decline.

#### 3.1.1. Tap Water Contamination

Contamination of water sources by antibiotics is a widespread global environmental concern, with numerous reports documenting their presence in both freshwater sources and drinking water systems. Although the situation varies across countries and water sources, previous research has confirmed that antibiotic residues in potable water are ubiquitous. Ben et al. found 58 antibiotics in tap water from ten Chinese cities (average 182 ng/L, median 92 ng/L), a source aquarists often use untreated [106]. In a separate study, Voigt et al. documented the contamination of a drinking water reservoir by discharge from two sewage treatment plants, detecting macrolide antibiotics, such as clarithromycin (up to 0.60 μg/L), sulfamethoxazole (up to 0.40 μg/L), and trimethoprim (up to 0.39 μg/L), downstream of the treatment facilities [107]. Similarly, Vilca et al. found ciprofloxacin in Puno’s drinking water supply, sourced from Lake Titicaca, with mean concentrations of 188.1 ng/L during the dry season and 222.2 ng/L during the wet season [108]. Traditional water treatment facilities commonly employ processes such as coagulation, flocculation, sedimentation, filtration, and disinfection. However, Zhang et al. demonstrated that these conventional methods are ineffective at removing antibiotics from water [109]. Aquarists routinely use dechlorinated tap water for aquarium maintenance, including initial filling, evaporation compensation, and regular water changes. To limit the progression of antibiotic resistance from tap water residues, aquarists could use household water purifiers (e.g., activated carbon filters) or, where feasible, boil tap water prior to use to partially reduce antibiotic levels and decrease selective pressure in aquaria [110]. While advanced filtration methods like reverse osmosis (RO) can remove over 95% of antibiotics, these systems are rarely implemented in municipal water treatment facilities [111]. Using RO water is also an expensive option for household aquarists. Additionally, antibiotics such as oxytetracycline have been detected in tap water at levels as high as 0.6 ng/mL, raising concerns about continuous selecting for ARB in the aquatic environments of ornamental fish [112]. Therefore, antibiotic concentrations in tap water are likely to remain unchanged after standard water treatment processes. Consequently, antibiotics present in tap water can continuously enter household aquaria unnoticed through these regular maintenance practices.

Therefore, even though aquarists do not administer any antibiotics, trace amounts of antibiotics would still be present in aquaria at low concentrations through initial water filling, routine water replacement and replenishment. Research suggests that antibiotic mixtures at subtherapeutic levels can still sustain antibiotic resistance [113]. As a result, ARB associated with ornamental fish are continuously exposed to antibiotics and persist in household aquaria. However, there are more means to introduce antibiotics.

#### 3.1.2. Antibiotics in Fish Feed

The situation becomes even more complicated when considering the role of diet in ornamental fish systems. Commercial fish feeds, which serve as the primary energy source for most ornamental fish, can be a significant yet hidden route for the introduction of antibiotics into household aquaria. While direct evidence for antibiotic addition to ornamental fish feed is currently limited, studies on fish feed for food fish have documented this practice. For example, Chen et al. detected three antibiotics—sulfamethoxazole, enrofloxacin, and erythromycin—in fish feed samples from their studied farms [114]. In a subsequent study, Chen et al. found at least six different antibiotics in feed samples collected from four fish farms, with ciprofloxacin being one of the major antibiotics detected [115]. These findings clearly demonstrate that fish feed serves as a primary route for antibiotic administration.

Antibiotics in fish feed have been suggested to improve feeding rates in fish, potentially incentivizing their inclusion in feed formulations [116]. In an experiment conducted by the authors, approximately 50 µg/kg of erythromycin was detected in fish feed pellets intended for groupers (unpublished data), despite no mention of this antibiotic in the ingredient list. Given these findings and lax regulation of feed additives, similar practices are likely to extend to ornamental fish feed. Manufacturers may intentionally add antibiotics to reduce disease outbreaks and boost fish growth and survival, enhancing their product’s reputation. In aquaria, ornamental fish often consume commercial feed pellets, but any uneaten feed can leach residual antibiotics into the water. This continuous release of antibiotics not only exposes fish to subtherapeutic levels but also promotes the selection of resistant bacteria within the tank’s biofilm. Over time, the persistent low-level presence of these antibiotics in the aquarium environment can facilitate the development and maintenance of antibiotic-resistant microbial populations.

Observations in the food fish aquaculture sector, combined with the largely unregulated nature of feed additives in ornamental fish production, suggest that similar practices may prevail in this sector. Many brands available in the commercial fish feed industry typically carry warning labels such as ‘not intended for human consumption’ or ‘not intended for food fish or animals’. Some products even emphasize characteristics like “disease resistance” or “bacteria inhibiting”. Because ornamental fish are not primarily designated for human consumption, the regulation and monitoring of feed additives are far less stringent compared to those for food fish. This regulatory gap not only leaves room for undeclared antibiotics in fish feed pellets but also poses a risk when unconsumed feed releases residual antibiotics back into aquarium water, continuously exposing resident fish and promoting the selection of resistant bacteria within tank biofilms.

The use of live fish as prey for piscivorous ornamental fish may serve as a significant source of antibiotics in aquaria. Piscivorous fish are those that primarily feed on other fish, and they are popular among aquarists for their predatory behavior and striking appearance. Common examples include arowana (*Scleropages formosus*), oscar (*Astronotus ocellatus*), alligator gar (*Atractosteus spatula*), redtail catfish (*Phractocephalus hemioliopterus*), and iridescent shark (*Pangasianodon hypophthalmus*). It is rather common for aquarists to feed these species with smaller, low-cost ornamental fish, such as goldfish, platies, and larvae of Chinese carps. As those feeder fish are also ornamental species, they may carry significant amounts of antibiotics. As a result, the practice of live feeding could unintentionally introduce antibiotics into aquarium systems, contributing to their accumulation and the potential development of antibiotic resistance in piscivorous fish and their environments.

The bioaccumulation of antibiotics in fish has been extensively documented in the food fish aquaculture sector, where studies have shown that antibiotics can be found in various tissues. For example, it has been observed that ciprofloxacin and enrofloxacin can be transported easily from plasma and muscle [114]. Li et al. observed that at least two antibiotics can be detected in the muscles of cultivated produces [117]. Our previous studies have pointed out that ornamental fish in retail stores are exposed to multiple antibiotics [28,29]. Similar studies on ornamental fish are lacking, but the frequent use of antibiotics for prophylactic purposes and disease treatment in ornamental aquaria suggests that these fish may also accumulate significant concentrations of antimicrobial compounds in their tissues.

When piscivorous ornamental fish consume antibiotic-laden feeder fish, biomagnification may occur, leading to localized high concentrations of antibiotic residues, particularly within the digestive tract. This creates a scenario in which the gut microbiota of piscivorous fish is exposed to elevated levels of multiple antibiotics, potentially accelerating the selection and proliferation of ARB and ARGs within their microbial communities. This pathway of bioaccumulation and trophic transfer through the aquarium food chain represents an often-overlooked mechanism of antibiotic exposure in ornamental fish systems, although studies investigating the trophic transfer of ARB and ARGs in these organisms remain limited.

#### 3.1.3. Intentional Antibiotic Use

Veterinary-exclusive antibiotics, such as nitrofurans and malachite green, are readily obtainable in ornamental fish supply shops and are often marketed under various brand names or labeled as “fish medications” or “aquarium treatments”. Although direct evidence of their intentional use is limited, there is reasonable concern regarding the co-selection of antibiotic resistance, whereby the use of one antibiotic may promote resistance to multiple drugs through shared resistance mechanisms.

E-commerce platforms offer a convenient means of purchasing antibiotics [118]. By early 2025, this issue persists and has become increasingly complex. Through the use of specific search terms and keyword combinations, individuals can easily obtain a wide range of antibiotics—including tetracycline, oxytetracycline, doxycycline, ciprofloxacin, erythromycin, and amoxicillin—in large quantities and without prescriptions, even though some of these drugs are classified as over-the-counter (OTC) medications. The problem is further exacerbated by the growing shift in antibiotic sales to personal social media platforms, making it even more difficult to track and monitor usage patterns. Sellers exploit marketing tactics, using detailed product photographs and terms such as “antibacterial”, “anti-inflammation” to target common fish ailments. These strategies effectively bypass regulatory scrutiny by framing the products as fish-specific treatments rather than conventional antibiotics.

Unrestricted access to these antibiotics through physical outlets and online channels has contributed to their widespread abuse in the ornamental fishkeeping industry. Moreover, these antimicrobials can harm beneficial bacteria in aquarium filtration systems, destabilizing the ecosystem and perpetuating recurring issues. In fact, it has been suggested that antibiotics are toxic to fish [119,120]. Prolonged exposure could compromise fish health by disrupting physiological processes and weakening immune responses, ultimately reducing overall viability.

While aquarists are less likely to use antibiotics prophylactically, many are attracted by advertisements and the absence of veterinary prescription requirements to purchase these drugs for treating diseases. Consequently, aquarists often rely on their own experiences or inaccurate instructions when administering these products, which further exacerbates the potential for inappropriate and excessive usage. This self-directed approach increases the risk of abuse and further complicates efforts to monitor and regulate antibiotic use within the ornamental fishkeeping industry.

### 3.2. Pathways of Human Exposure

#### 3.2.1. Bioaerosols from Aeration

Aeration in aquaria can spread ARB beyond water via bioaerosols. Recent advances in aerosol research have revealed a previously underappreciated transmission pathway for ARB and ARGs in indoor environments. ARGs have been detected in various aerosol-generating facilities, including waste treatment plants, livestock farms, and biogas digesters [121,122]. Indoor settings are particularly concerning because elevated ARG levels can have serious implications for occupant health. For example, studies have shown that indoor wet markets can exhibit ARG levels (5.5 copies/m^3^) that are up to thirty times higher than those outdoors [123]. Zhao et al. further confirmed that indoor environments contain significantly elevated levels of culturable bacteria, ARGs, and bacterial 16S rRNA copies [124]. Continuous exposure to these bioaerosols may alter the human microbiome. Yang et al. demonstrated this by identifying clear parallels in microbial diversity and ARG distribution among chicken coops, poultry farm aerosols, and nasopharyngeal samples from farm workers [125].

In this context, ornamental aquaria may inadvertently contribute to the indoor burden of ARB and ARGs through their standard aeration practices. These systems typically employ air pumps and air stones to maintain dissolved oxygen levels—an essential process for tanks housing larger fish or higher stocking densities. However, the aeration process generates bioaerosols through bubble formation and bursting. The use of higher-powered pumps and air stones that generate smaller bubble sizes, although preferred for enhanced gas dissolution, may inadvertently increase bioaerosol production [126]. Similarly, ornamental fish farms, which use even higher power pumps, could result in more serious health risk to workers, creating potential occupational health risks.

Cases of infections include *A. hydrophila* from an aquarium air pump infecting a child with cystic fibrosis and causing peritonitis linked to a goldfish [127,128]. Additional studies have found that viable *A. salmonicida* can be recovered from experimentally generated aerosols at a distance from the source, while *P. aeruginosa* may persist in bioaerosols for extended periods [129,130]. Transmission of NTM through aerosolized water exposure has also been reported, underscoring aeration as a hidden indoor health risk [131].

#### 3.2.2. Direct Contact and Environmental Exposure

Routine tank maintenance exposes aquarists directly to resistant pathogens. In addition to airborne transmission, direct contact with aquarium water represents another significant pathway for exposure to zoonotic bacteria, ARB, and ARGs. Routine tasks like water changes and tank cleaning expose aquarists to aquarium water. Moreover, the disposal of this water through household facilities like washrooms or kitchen sinks can lead to contamination of areas that are frequently used, thereby increasing exposure risks for household members.

Direct water contact has been established as a key route for transmitting zoonotic pathogens from ornamental fish systems. Although cases specifically linked to ornamental fish remain relatively limited, recent events underscore potential hazards. A notable example is the endemic outbreak of invasive Group B Streptococcus infection, where contact with contaminated water and infected fish during routine maintenance—often with bare hands—resulted in infections among 91 otherwise healthy individuals [132]. This outbreak underscores the risks of direct exposure even among individuals without pre-existing health conditions. Balagué et al. reviewed non-tuberculous mycobacterial infections of hands and found that many cases were related to aquatic environments [133].

The dangers associated with direct water contact are further amplified by current aquaculture practices. Research has demonstrated that the use of antibiotics in ornamental fish systems can select for pathogenic bacteria, potentially leading to the emergence of more virulent or resistant strains [29]. The combination of inevitable water contact during maintenance and the presence of these potentially resistant pathogens creates a significant public health concern that warrants careful consideration and preventive measures.

#### 3.2.3. Zoonotic Risks

Zoonotic diseases link fish pathogens to human illness. Ornamental fish are associated with a range of zoonotic pathogens that pose significant health risks to humans. *Aeromonas* spp.—particularly *A. hydrophila*, *A. caviae*, and *A. veronii*—are recognized as important pathogens in aquatic environments. These bacteria can cause various infections, including gastroenteritis (which may range from mild diarrhea to severe dysentery), especially in immunocompromised individuals [134,135]. Studies have noted that *A. veronii* is frequently isolated from patients with gastrointestinal infections, emphasizing its role as a significant enteric pathogen [136,137].

In addition to gastrointestinal illnesses, *Aeromonas* species are implicated in skin and soft tissue infections. Exposure to contaminated water or breaches in the skin can lead to severe conditions such as necrotizing fasciitis—particularly linked to *A. caviae*—which can progress rapidly and cause systemic complications [138,139]. Bacteremia, the presence of bacteria in the blood, has also been associated with *Aeromonas* infections, especially in patients with weakened immune systems [135,140]. Beyond these, *Aeromonas* spp. has been connected with respiratory and urinary tract infections as well as more severe manifestations like meningitis and septic arthritis [140,141]. Notably, *Aeromonas* spp. can aerosolize during aquarium operations, posing a risk of transferring resistance genes—including those for last-resort antibiotics like colistin—to household aquarists [142,143]. This aerosolization capability increases the likelihood of exposure through inhalation, which further emphasizes the need for rigorous preventive measures among those who maintain ornamental fish systems.

Other pathogens associated with ornamental fish include various *Mycobacterium* spp. (e.g., *M. marinum*), which are known to cause cutaneous infections following exposure during aquarium maintenance [144,145]. Reports indicate that 49–84% of *M. marinum* cases are associated with exposure to ornamental fish [146,147]. *Vibrio* spp.—such as *V. cholerae*, *V. parahaemolyticus*, and *V. vulnificus*—are also present in aquatic environments. These bacteria can cause illnesses ranging from gastroenteritis and wound infections to septicemia, particularly in individuals with underlying health conditions [148,149,150,151]. In addition, species such as *V. alginolyticus* and *V. harveyi* have been implicated in cellulitis and other infections associated with ornamental fish systems [152]. Similarly, *Streptococcus iniae*, a pathogen affecting both fish and humans, is known to cause a range of infections from cellulitis to septicemia following exposure to infected ornamental fish [153].

To minimize risks from ARB to farm staff and aquarists, water treatment devices, such as UV sterilizers or ozone generators, can decrease bacterial loads in tanks, reducing exposure potential. Direct contact with tank water should also be avoided by using tools such as siphons or wearing gloves, particularly when open wounds are present. Although no studies specifically evaluate bioaerosol risks in ornamental fish settings, personal protective equipment (PPE), such as masks, may be beneficial during aeration or water changes–especially in poorly ventilated areas or with high-powered aerators–to limit inhalation of resistant pathogens. The FDA advises against washing frozen chicken under running water to prevent bacterial spread through splashed droplets; similarly, discarding tank water could disperse ARB onto surrounding surfaces, sinks, or toilets [154]. Careful disposal practices, such as using sealed containers, combined with thorough cleaning of affected areas, are essential to lower contamination risks.

Collectively, these findings underscore that both direct contact with aquarium water and the aerosolization of contaminated water are routes for zoonotic pathogens, in particular ARB and ARGs, to reach humans. Acknowledging these diverse transmission pathways highlights the critical need for robust aquarium management practices, refined water disposal protocols, and enhanced regulatory oversight to mitigate the associated health risks. Moreover, further research is needed to elucidate the mechanisms driving resistance selection and to fully understand the implications for human health.

## 4. Challenges and Management in the Industry

Despite its economic and global reach, the ornamental fish industry lacks research on antibiotic use, resistance, and sustainability. This knowledge gap is especially concerning given the industry’s heavy reliance on antibiotics for disease management and the potential consequences of unregulated antibiotic application in the industry. To address this gap, several global initiatives have been launched to curb antibiotic resistance, but not all of them cover the ornamental fish sector.

The “Global Action Plan on Antimicrobial Resistance” (GAP-AMR) emphasizes several strategic objectives, including strengthening the knowledge and evidence base through surveillance and research, optimizing the use of antimicrobial medicines in animal health, reducing the incidence of infections, and developing sustainable investment strategies [155]. However, within the aquaculture sector, only 60% of World Health Organization (WHO) member states have developed a ‘National Action Plan on Antimicrobial Resistance’ (NGP-AMR) aligned with the One Health perspective, which emphasizes addressing AMR across the entire supply chain [156]. As a subsector of aquaculture, the ornamental fish industry has received even less attention regarding the spreading of antibiotic use and resistance, leaving significant gaps in our understanding of antibiotic use and the mechanisms driving resistance spreading.

The One Health approach is particularly well-suited to address the intertwined challenges of antibiotic abuse and resistance spreading in this context, as it involves multiple stakeholders, including the health of fish, humans, and the environment. Government agencies and regulatory authorities must take a leading role in designing and implementing independent accreditation schemes. Such measures could help decouple the benefits of human and environmental health from the risks posed by antibiotic residues and resistance determinants that have emerged in this rapidly expanding yet unregulated sector.

Considering these issues, we aim to explore the current challenges and future prospects in the ornamental fish industry, focusing on the difficulties in surveillance and management, the spread of antibiotic resistance, and the resulting environmental impacts. Addressing these challenges is crucial for ensuring long-term sustainability of the industry, mitigating the risks associated with antibiotic resistance, and aligning the sector with global antimicrobial stewardship efforts.

### 4.1. Surveillance and Management Challenges

Current legislation and guidelines regarding antibiotic use in the ornamental fish industry are rather limited. Authorized sources for accessing necessary medications for ornamental fish, such as the European Medicines Agency (EMA) and the U.S. FDA, enhance transparency in drug use, thereby ensuring safer application and reducing the risk of unregulated and illegal drug use in minor species (including ornamental fish) [157,158]. The European Union (EU) has implemented stringent guidelines and legislation covering the ornamental fish industry, by restricting it so that only dimetridazole, flavophospholipol, oxytetracycline, sulfadoxine and trimethoprim can be used for bacterial infections in ornamental fish [157]. In the United Kingdom, legislations, including the Animal Welfare Act. 2006 (AWA), the Veterinary Surgeons Act. 1966 (VSA) and the Veterinary Medicines Regulations 2013 (S.I. 2013/2033; VMR), work together to regulate animal welfare and the use of veterinary medicines [159,160,161]. Specifically, the AWA mandates that the basic needs of all animals, including fish, be met by law. However, because the provisions of the VSA do not cover fish, non-veterinary personnel are permitted to diagnose and treat fish diseases. The VMR, meanwhile, sets the legislation framework for the use of veterinary medicines and medicated feed in fish. In Asia, the Japanese government regulates the use of antibiotics in fish farming, including in ornamental species such as koi carp, by requiring approvals from the authorities. Applications must provide documents detailing the antimicrobial spectrum of the product, results of antimicrobial susceptibility tests on recent field isolates of target, indicator, and zoonotic bacteria, as well as resistance acquisition test results [162]. In China, although only chemical and parasitic treatments are officially recommended for controlling infections in ornamental fish farming, a wide variety of antibiotics were still detected in practice [32,163]. Over 90% of carriage water samples of ornamental fish contained residues of various antibiotic classes, including chloramphenicol, fluoroquinolones, nitrofurans, and tetracyclines, highlighting deficiencies in border inspections for antibiotic residues in imported ornamental fish [30]. Taken together, current drug indexing and surveillance programs are insufficient to meet industry needs. The shortlisted drugs leave farmers and retailers with few legal treatment options, forcing them to resort to a broader range of antibiotics to prevent and control infections, thereby exacerbating antibiotic abuses and resistance in the industry.

Additionally, absence of centralized record-keeping in the ornamental fish industry further complicates the monitoring of antibiotic usage, tracking of resistance trends, and enforcement of biosecurity measures, while also limiting traceability throughout the supply chain. Given that ornamental fish are bred, transported, and sold through multiple intermediaries across different countries, this fragmented supply chain poses a significant challenge to effective surveillance on antibiotic use and resistance trends [21]. Similar to practices observed in the food fish aquaculture sector, where antibiotics are used both prophylactically and therapeutically, the involvement of multiple independent stakeholders operating under varying regulations makes it difficult to track antibiotic abuse [23]. Consequently, the ornamental fish industry creates an environment that facilitates the spread of antibiotic resistance.

### 4.2. Environmental Impact and Resistance Spreading

Inadequate surveillance and poor management systems in the ornamental fish industry have led to antibiotic abuse, which, in turn, significantly contributes to antibiotic resistance spreading and environmental pollution. Ornamental fish are typically kept in water ranging from 25 °C to as high as 30 °C for certain tropical species, with pH values between 6 and 8. These environmental parameters significantly influence the distribution and persistence of antibiotics, thereby affecting their ecological impact. For example, tetracyclines remain relatively stable at near-neutral pH conditions (6.5–7.5). Loftin et al. observed that the half-life of tetracycline dropped from 107 days at pH 7 and 20 °C to only 10 days at pH 5 and 30 °C, demonstrating how temperature and pH combine to accelerate degradation [164]. Likewise, Doi and Stoskopf reported that oxytetracycline degrades more rapidly at both acidic and alkaline extremes, but persists longer at neutral pH [165]. Other antibiotic classes also exhibit varying stability under different conditions. For example, sulfamethoxazole is relative stable for months in water at neutral to slightly acidic pH but degrades more quickly at higher temperatures and under alkaline conditions [166]. Similarly, amoxicillin becomes highly unstable at pH levels below 6 or above 8, reducing its persistence from days to mere hours [167].

Antibiotic residues have been constantly detected in the wastewater and carriage water of ornamental fish, demonstrating the frequent and unregulated use of antibiotics in the industry [28,29,168]. Such indiscriminate practices have fostered the emergence of ARB and compromised water quality [13,14]. Studies have demonstrated the dissemination of veterinary antibiotics from aquaculture and animal husbandry with ARG occurrence into the surrounding environments [169,170]. Likewise, extensive use of tetracyclines, fluoroquinolones, sulfonamides, and nitrofurans, often without veterinary oversight, raises concerns regarding antibiotic residues in carriage water, the potential transmission of resistance to human handlers, and broader environmental contamination. Without improved regulatory oversight and enhanced research efforts, the role of the ornamental fish industry in contributing to the global antibiotic resistance crisis may remain largely underestimated.

Antibiotics in aquatic environments not only contribute antibiotic resistance but also disrupt essential biogeochemical cycles, such as the nitrogen cycle, in carriage water. Research reported that sulfonamide concentrations as low as 50 ng/L can reduce denitrification rates by 20–30%, corresponding to a 39–53% decrease in the abundance of denitrifying functional genes [171]. These disruptions can lead to the accumulation of nitrogen in aquatic environments, exacerbating eutrophication and significantly deteriorating water quality. In ornamental fish farming, such compromised conditions further challenge efforts to maintain fish welfare, potentially leading to reduced growth and survival rates during transportation and at retail outlets [172]. Thus, the ecological impacts of antibiotic abuse in the ornamental fish industry extend beyond the development of antibiotic resistance, ultimately undermining fish welfare and threatening the long-term sustainability of the industry.

### 4.3. Strategic Regulatory and Management Approaches

To address the growing concerns of antibiotic resistance in the ornamental fish industry, a multi-faceted approach is indispensable. This should integrate education, regulation, and international cooperation to mitigate the threats posed by antibiotic abuse and resistance. Given the heavy reliance on antibiotics for disease management of the industry, it is important to implement effective strategies that ensure prudent use, limit environmental pollution, and support sustainable practices.

#### 4.3.1. Education and Awareness Initiatives

Managing antibiotic use on a scientific basis begins with comprehensive training programs for all stakeholders, including aquarists, retailers, and other industry professionals. Training programs should emphasize proper antibiotic selection, accurate dosing, and effective administration techniques to minimize abuse. Moreover, these programs must address the consequences of indiscriminate antibiotic use, such as the emergence of resistance stains of *Aeromonas* spp., which have already led to significant losses in the industry [173]. By raising awareness about antibiotic resistance, these initiatives encourage adherence to responsible treatment practices, ensuring that antibiotics remain effective when genuinely needed. In addition, developing comprehensive best practice guidelines for disease management, responsible antibiotic use, and proper handling techniques is crucial. For example, the aquaculture industry (including koi carp) in Japan has successfully implemented biosecurity measures and restricted antibiotic use through guideline-based disease control framework, a model that could be adapted for the global ornamental fish industry [162]. Finally, public education must also be prioritized so that the general community is informed about the potential zoonotic risk, especially the exposure to multidrug-resistant bacteria presenting in the carriage water of ornamental fish.

#### 4.3.2. Regulatory Measures

Complementing educational efforts, regulatory measures are vital to ensure that industry practices align with established standards. Local regulatory bodies should conduct routine inspections to monitor antibiotic use in ornamental fish facilities, with robust enforcement mechanisms in place to detect unauthorized applications. National surveillance programs, such as the European Surveillance of Veterinary Antimicrobial Consumption (ESVAC), have systematically tracked antibiotic use and resistance trends to promote the responsible use of antibiotics in aquaculture [174,175,176,177,178,179]. A similar approach should be adopted globally within the ornamental fish industry. Ongoing monitoring efforts must also focus on tracking antibiotic resistance patterns in both the ornamental fish and their environments. Recent studies have highlighted the presence of ARB and significant antibiotic resides in imported ornamental fish and carriage water, emphasizing the need for enhanced surveillance [28,29,30].

To further strengthen oversight, mandatory reporting systems should be implemented to establish a centralized database for tracking antibiotic use and resistance trends. Additionally, guidelines for the proper disposal of wastewater and contaminated materials must be developed to prevent environmental spreading of ARB and ARGs. Licensing systems for all stakeholders should also be introduced, with requirements that include proof of antibiotic stewardship straining, adherence to biosecurity measures, and compliance with standardized disease management protocols. A globally recognized antibiotic use framework is vital for uniform regulations across countries. For example, the Japanese government has implemented strict antibiotic policies in aquaculture, including the ornamental fish industry, that require prescriptions and impose rigorous monitoring [162]. This could serve as a benchmark for developing similar policies within the industry. International collaboration remains essential for harmonizing treatment protocols, residue limits, and surveillance programs, thereby fostering a cohesive global response to antibiotic resistance challenges.

#### 4.3.3. Alternative Disease Prevention Strategies

While regulatory oversight and education are vital, it is equally important to explore alternative disease prevention strategies that reduce dependency on antibiotics. Research into other sustainable solutions, such as vaccination, probiotics, and other immunostimulants, offers promising avenues for disease management in the ornamental fish industry. By integrating these methods into routine husbandry and management practices, stakeholders can significantly minimize their reliance on antibiotics while improving fish health and overall biosecurity.

Adherence to good aquaculture practices, including optimizing stocking density and monitoring water quality, plays a critical role in disease prevention. Poor management practices, such as overcrowding and suboptimal water conditions, increase the incidence of infections, thereby triggering higher antibiotic use. In contrast, well-managed systems help mitigate stress and disease outbreaks, reducing the need for antibiotics, and ultimately improving fish welfare. It is important to note that, although antibiotics can offer short-term therapeutic benefits, their indiscriminate use may produce toxic effects in fish. Prolonged exposure can weaken fish immunity, making them more susceptible to infections, while also promoting microbial resistance [180,181]. Moreover, antibiotics that are only partially metabolized or excreted tend to accumulate in water systems, further contributing to resistance gene transfer and disrupting microbial communities essential for fish health.

The use of feed supplements has been widely studied in fish over the past decades. Various feed supplements have been studied as alternatives to antibiotics in fish aquaculture, including probiotics, prebiotics, natural antioxidants, plant-based ingredients, and fermented products, which can improve growth and feed efficiency, and enhance immunity and organ health [182,183]. For example, probiotics are increasingly considered as a safer and more environmentally friendly alternative to antibiotics in the aquaculture sector. They have demonstrated multiple benefits, including improved larval development, growth promotion, immune stimulation, disease control, and stress resistance [184]. Additionally, probiotics improve water quality by stabilizing microbial communities and decreasing reliance on antibiotics. Research has also shown that probiotics can also mitigate the adverse effects of antibiotic-induced microbiome disruptions, thereby reducing fish mortality and strengthening disease resistance [185,186]. Several probiotic strains, including *Lactobacillus*, *Bacillus*, and *Saccharomyces* species, have been tested in ornamental fish with promising results. For example, *Bacillus subtilis* has been shown to improve disease resistance in sailfin molly by improving gut microbiota stability, while *Lactobacillus rhamnosus* supplementation in goldfish led to enhanced immune responses and reduced bacterial infections [187,188]. Widespread use of probiotics in ornamental fish facilities offers a practical, sustainable alternative to antibiotics

Innovative water treatment technologies, such as biofilter adsorption, activated carbon filtration, and ultraviolet (UV) sterilization, have been explored as potential solutions for removing residual antibiotics from water systems [189,190]. Studies have demonstrated that activated carbon filtration methods allow up to 89% removal of antibiotics [191]. Other wastewater treatment technologies, such as advanced oxidation processes (AOPs) and membrane bioreactors, have shown promising results in effectively removing up to 88% of antibiotics and ARGs from aquatic environments [192]. Gayatri et al. investigated photocatalysis with UV light for degrading antibiotics and found that UVC light enhances the breakdown of antibiotic compounds [190]. Integrating these water treatments into ornamental fish facilities, where feasible, can reduce contamination and maintain water quality.

The ornamental fish industry must embrace a holistic approach to disease management that balances education, regulation, and sustainable disease prevention strategies. By integrating these sustainable practices, the industry can align with global efforts to combat antibiotic resistance, minimizing reliance on antibiotics while enhancing fish welfare and promoting environmental sustainability. This approach not only improves animal health but also addresses the interconnected challenges of human, animal, and environmental health, fostering a unified strategy to tackle antibiotic resistance to all levels.

## 5. Conclusions

This review explores current insights into antibiotic use, resistance, and the associated regulatory and management frameworks within the ornamental fish industry. This sector constitutes a notable yet frequently overlooked contributor to the global antibiotic resistance crisis. The primary threat stems from unregulated antibiotic use, persistent residues in carriage water, and the emergence of multidrug-resistant bacteria across the supply chain. Such unchecked practices accumulate residues, fuelling the spread of ARB and ARGs, endangering fish, human health, and aquatic environments through diverse pathways. Despite mounting evidence of antibiotic resistance in this industry, significant knowledge gaps persist concerning its long-term effects on fish welfare, human health, and environmental sustainability. Tackling antibiotic abuse in ornamental fish demands a thorough grasp of the interplay among fish, their microbial communities, and their habitats. Future research should focus on evaluating transmission risks, assessing antibiotic impacts on fish health and environmental microbiota, and devising sustainable strategies to curb resistance emergence and spread, alongside a bolstering of regulatory oversight.

Alternative disease management approaches—such as probiotics, immunostimulants, and advanced water treatment technologies—present viable options to lessen antibiotic dependence. These methods enhance fish welfare, promote environmental sustainability, and protect public health. Moreover, global cooperation among regulatory bodies, researchers, and industry stakeholders is vital to standardize regulations and foster responsible antibiotic stewardship. Yet, the absence of comprehensive, uniform data on antibiotic use and resistance trends remains a critical barrier. This gap impedes a full evaluation of the scale of antibiotic use and resistance dissemination, highlighting the urgent need for systematic reporting and surveillance systems.

To confront these challenges, we advocate integrating the ornamental fish industry into the One Health framework to address antibiotic abuse and resistance holistically. This approach targets three key areas: (1) the health and welfare of ornamental fish; (2) human health risks, particularly from direct and indirect exposure to ARB; and (3) the integrity and function of aquatic ecosystems, encompassing habitats and ecological processes. Embracing the One Health perspective underscores the necessity for standardized guidelines, robust surveillance, and precise legislation within this industry. Coordinated efforts should identify high-risk practices and resistance hotspots to inform evidence-based policies.

A comprehensive strategy—merging advanced research, innovative alternatives, and stringent oversight—is crucial to protect fish welfare, sustain the ornamental fish trade, and shield public health from the escalating global threat of antibiotic resistance.

## Figures and Tables

**Table 1:** Popular ornamental fish family and species.

Family	Species	Common Name
Characidae	*Hyphessobrycon megalopterus*	Black phantom tetra
	*Paracheirodon innesi*	Neon tetra
Cichlidae	*Astronotus ocellatus*	Oscar
	*Pterophyllum altum*	Angelfish
Cyprinidae	*Carassius auratus*	Goldfish
	*Cyprinus rubrofuscus*	Koi
Gasteropelecidae	*Gasteropelecus sternicla*	Hatchetfish
Loricariidae	*Ancistrus spinosus*	Armored catfish
	*Hypostomus plecotomus*	Suckermouth catfish
Poeciliidae	*Poecilia reticulata*	Guppy
	*Xiphophorus maculatus*	Platies

**Table 2:** Antibiotic residues detected in carriage water of ornamental fish across various regions and countries.

Antibiotic Class		Region/Country	Detected Antibiotics	References
Antibiotic class	Amphenicol	Mainland China, PRC	Chloramphenicol	[32]
		The Netherlands	Chloramphenicol	[30]
	Macrolide- lincosamide-streptogramine	Hong Kong, PRC	Clarithromycin, roxithromycin	[29]
	Nitrofuran	The Netherlands	Ciprofloxacin, enrofloxacin, oxalinic acid	[30]
	(Fluoro) quinolone	Germany	Enrofloxacin	[31]
		Mainland China, PRC	Ciprofloxacin, enrofloxacin, norfloxacin	[32]
		The Netherlands		[30]
		Hong Kong, PRC	Ciprofloxacin, enrofloxacin, oxalinic acid	[28,29]
	Sulfonamide /Trimethoprim	Mainland China, PRC	Sulfadimethoxine, sulfadiazine, sulfameter, sulfamethazine, sulfathiazole	[32]
	Tetracycline	Germany	Tetracycline	[31]
		Mainland China, PRC	Chlortetracycline, oxytetracycline, tetracycline	[32]
		The Netherlands		[30]
		Hong Kong, PRC	Chlortetracycline, doxycycline, oxytetracycline, tetracycline	[28,29]

## Data Availability

No new data were created or analyzed in this study.
